# Implementation of an Mpox Vaccination Program at a Large Sexual Health Clinic in the Bronx—Lessons in Vaccine Equity

**DOI:** 10.1093/ofid/ofad544

**Published:** 2023-10-31

**Authors:** Usama Irshad, Derek Bishop, Caroline Mullis, Justin Toro, Barry S Zingman, Eric A Meyerowitz

**Affiliations:** Department of Medicine, Montefiore Medical Center and Albert Einstein College of Medicine, Bronx, NewYork, USA; Albert Einstein College of Medicine, Bronx, NewYork, USA; Division of Infectious Diseases, Department of Medicine, Montefiore Medical Center, Bronx, NewYork, USA; Division of Infectious Diseases, Department of Medicine, Montefiore Medical Center, Bronx, NewYork, USA; Division of Infectious Diseases, Department of Medicine, Montefiore Medical Center, Bronx, NewYork, USA; Division of Infectious Diseases, Department of Medicine, Montefiore Medical Center, Bronx, NewYork, USA

## Abstract

Mpox caused a global outbreak in 2022. Among 249 people who received mpox vaccination at a sexual health clinic in the Bronx, New York, those with private vs public insurance were more likely to complete the series. No mpox cases were seen during follow-up at a median 121 days (IQR, 97–139).

Mpox, which has been endemic in parts of western Africa for >5 decades [1], caused a global outbreak starting in May 2022 primarily in the LGBTQ+ population, especially in gay, bisexual, and other sexual and gender minority men [2] (GBSGMM). In the United States, the smallpox vaccine (JYNNEOS; modified vaccinia Ankara–Bavarian Nordic) was deployed to provide cross-protection, since data from a previous mpox outbreak in Zaire (now the Democratic Republic of the Congo) suggested that smallpox vaccines were around 85% effective at preventing mpox [3, 4].

Mpox vaccine administration data in the United States highlight important health care disparities. While 63.9% of mpox cases in the United States have occurred in Black and Hispanic/Latinx individuals, these groups constitute just 20.8% and 11.3% of those who received at least 1 vaccine dose, respectively, as of 19 July 2023 [5, 6].

We describe our experience with the 2-dose JYNNEOS vaccine administered subcutaneously at a 28-day interval at The Oval Center at Montefiore (TOCM), Montefiore Medical Center's largest clinic for sexual health and LGBTQ+ care, in the Bronx, New York. TOCM was selected as one of the first community sites for rollout of mpox vaccination following expansion from New York City Department of Health clinics. TOCM serves a marginalized population, with 85.2% of the Bronx population identifying as Black and/or Hispanic/Latinx and 26.1% living in poverty (greater than the 15.6% and 10.9% in New York City and New York State, respectively) [7]. To understand the implementation of an mpox vaccine program at TOCM and to identify strengths and shortcomings to improve future similar programs, we analyzed our mpox vaccine administration data.

## METHODS

The cohort included all unique individuals who received at least 1 mpox vaccine from 1 August 2022 to 1 January 2023 at TOCM. At the time, vaccine eligibility included GBSGMM, people with multiple sex partners, or individuals at increased risk for mpox. While outreach was made only to established Montefiore patients, TOCM was listed as a vaccination site on the New York City Department of Health website, and eligible individuals outside Montefiore could call to request vaccination. No individuals who requested vaccination and met eligibility criteria were turned away.

Data, extracted by manual chart review of electronic medical records, included patient demographics, total mpox vaccine doses received, confirmed bacterial sexually transmitted infections between 1 January 2022 and 1 January 2023, date of any positive mpox polymerase chain reaction tests, antiretroviral therapy prescriptions and most recent CD4 cell count and HIV-1 viral load before 1 January 2023 for people living with HIV (PLWH), and prescriptions for preexposure prophylaxis (PrEP) against HIV for people living without HIV (PLWoH). Insurance status was classified as public if primary insurance was Medicaid, Medicare, or an AIDS drug assistance program or PrEP patient assistance program or private if a commercial or union plan. Patients were “established” if they received primary or sexual health care within Montefiore with at least 1 relevant provider visit note in the electronic medical record (Montefiore used paper charts before 2015); otherwise, they were “unestablished.” Among those who tested positive for mpox, we collected detailed information, including symptoms and severity, whether tecovirimat was administered, and the timing of mpox presentation in relation to vaccine doses administered.

Categorical data were summarized as frequencies and percentages. Continuous variables were summarized as mean with SD or median with IQR. Bivariate associations between established patient status and variables of interest and between the number of doses received and the same variables were evaluated by chi-square tests and Student *t* test with equal variance. Multivariable logistic regression was performed to evaluate association between receipt of 2 doses of vaccine and variables of interest. The initial model for receipt of 2 vs 1 vaccine dose included age, race/ethnicity, whether established or unestablished at Montefiore, and insurance type a priori. Statistical assumptions of the model were evaluated and met. Only associations with 2-sided *P* values <.05 were considered statistically significant. All statistical analyses were performed on Stata (version 17.0; StataCorp).

### Patient Consent Statement

The study was approved by the Albert Einstein College of Medicine Institutional Review Board (2022-14651). Informed patient consent was not required given the minimal risk and retrospective study design.

## RESULTS

An overall 249 people received at least 1 mpox vaccine at TOCM between 1 August 2022 and 1 January 2023, with a median follow-up of 121 days (IQR, 97–139 days) through 1 January 2023. In total, 206 (82.7%) were established at Montefiore and 43 (17.3%) were unestablished (Table 1). In bivariate analysis, unestablished patients were more likely to have private insurance (73.5% vs 40.2%, *P* < .001) and less likely to be Black (22.7% vs 38.5%, *P* < .01) or Hispanic/Latinx (27.3% vs 40.6%, *P* < .01), and a larger percentage of established patients were PLWH (53.4% vs 15.8%, *P* < .01).

**Table 1.** Characteristics of a Cohort Receiving Mpox Vaccine Doses at a Large Sexual Health Clinic in the Bronx, New York, Between 1 August 2022 and 1 January 2023 (N = 249)

	No. (%)			No. (%)		
	Established ^a^ (n = 206)	Unestablished ^b^ (n = 43)	*P* Value	2 Vaccine Doses (n = 197)	1 Vaccine Dose (n = 52)	*P* Value
Age, y^c^	38.6 ± 11.6	37.3 ± 10.6	.50	39.4 ± 11.4	34.5 ± 10.7	<.01
Category			.72			.06
19–29	49 (23.8)	10 (23.3)		40 (20.3)	19 (36.5)	
30–39	78 (37.9)	20 (46.5)		78 (39.6)	20 (38.5)	
40–49	37 (18.0)	6 (13.9)		36 (18.3)	7 (13.5)	
≥50	42 (20.4)	7 (16.3)		43 (21.8)	6 (11.5)	
Insurance type						
Medicare	19 (9.2)	0		15 (7.6)	4 (7.7)	
Medicaid	90 (43.7)	9 (20.9)		70 (35.5)	29 (55.8)	
ADAP/PrEP-AP	14 (6.8)	0		10 (5.1)	4 (7.7)	
Private Insurance	81 (39.3)	25 (58.1)		95 (48.2)	11 (21.2)	
Uninsured	2 (1.0)	2 (4.7)		2 (1.0)	2 (3.8)	
Unknown insurance status	0	7 (16.3)		5 (2.5)	2 (3.8)	
Insurance status			<.001			<.01
Public^d^	122 (59.8)	9 (26.5)		95 (50.0)	36 (75.0)	
Private	82 (40.2)	25 (73.5		95 (50.0)	12 (25.0)	
Self-reported race and ethnicity						
Hispanic/Latinx	76 (36.9)	6 (14.0)		69 (35.0)	13 (25.0)	
Non-Hispanic Black/African American	72 (35.0)	5 (11.6)		60 (30.5)	17 (32.7)	
Non-Hispanic White	17 (8.3)	8 (18.6)		24 (12.2)	1 (1.9)	
Asian	4 (1.9)	3 (7.0)		7 (3.6)	0	
Native Hawaiian	1 (0.5)	0		0	1 (1.9)	
Multiracial	17 (8.3)	0		12 (6.1)	5 (9.6)	
Not specified/declined	19 (9.2)	21 (48.8)		25 (12.7)	15 (28.8)	
Race/ethnicity (bivariate analysis)^e^			<.01			.16
Hispanic/Latinx	76 (40.6)	6 (27.3)		69 (40.1)	13 (35.1)	
Non-Hispanic Black	72 (38.5)	5 (22.7)		60 (34.9)	17 (46.0)	
White	17 (9.1)	8 (36.4)		24 (14.0)	1 (2.7)	
Other	22 (11.8)	3 (13.6)		19 (11.1)	6 (16.2)	
Vaccine doses received			.41			
1	41 (19.9)	11 (25.6)				
2	165 (80.1)	32 (74.4)				
Established vs unestablished						.40
Established				165 (83.8)	41 (78.8)	
Unestablished				32 (16.2)	11 (21.1)	
HIV status			<.01			.72
PLWH	109 (53.4)	3 (15.8)		90 (50.8)	22 (47.8)	
PLWoH^f^	95 (46.6)	16 (84.2)		87 (49.2)	24 (52.1)	
Bacterial STIs in the last year^g^			.16			.44
No	123 (60.6)	9 (81.8)		102 (60.4)	30 (66.7)	
Yes	80 (39.4)	2 (18.2)		67 (39.6)	15 (33.3)	

Abbreviations: ADAP, AIDS drug assistance program; EMR, electronic medical record; PLWH, people living with HIV; PLWoH, people living without HIV; PrEP-AP, preexposure prophylaxis patient assistance program; STI, sexually transmitted infection.

^a^Established patients had at least 1 provider visit note at the Montefiore Medical Center within 10 years available in the EMR.

^b^Unestablished patients had no provider visit notes at the Montefiore Medical Center within the EMR within 10 years.

^c^Mean ± SD.

^d^Primary insurance was Medicaid, Medicare, or ADAP/PrEP-AP. Analysis with 7 unknown and 4 uninsured excluded.

^e^Race/ethnicity categories considered in bivariate analysis were Hispanic/Latinx, Black, White, or other. Analysis excludes 40 participants with unknown or unspecified race/ethnicity.

^f^Analysis excludes 26 without documented HIV testing.

^g^Microbiologically confirmed in the EMR between 1 January 2022 and 1 January 2023.

The cohort included 112 PLWH (all diagnosed before first mpox vaccine dose), 111 PLWoH, and 26 of unknown HIV status (Supplementary Table 1). In total, 239 (94%) self-identified as cisgender men, 191 (76.7%) as gay, and 217 (87.1%) were GBSGMM. Between 1 August 2022 and 1 January 2023, 111 (99.1%) PLWH were prescribed antiretroviral therapy, and 95 (85.6%) PLWoH were prescribed any form of PrEP. Between 1 January 2022 and 1 January 2023, 15 (13.4%) PLWH and 6 (5.4%) PLWoH had a new syphilis infection, defined as new positive treponemal antibody or a 4-fold rise in rapid plasma reagin. During the same period, 14 (12.5%) PLWH and 22 (19.8%) PLWoH had chlamydia, and 21 (18.8%) PLWH and 21 (18.9%) PLWoH had gonorrhea. Ten (8.9%) PLWH and 8 (7.2%) PLWoH had >1 bacterial sexually transmitted infection.

In total 197 (79.1%) received 2 vaccines doses and 52 (20.9%) received 1 (Table 1). Participants receiving 1 dose had a lower mean age than those receiving 2 (34.5 ± 10.7 vs 39.4 ± 11.4 years, *P* < .01). In a multivariable model adjusting for age, race/ethnicity, whether established or unestablished at Montefiore, and insurance type, those who received 2 doses were around 3 times more likely to have private insurance as compared with those who received 1 (adjusted odds ratio, 3.10 [95% CI, 1.28–7.49]; *P* < .01; Supplementary Table 2). Sixty-nine (84.1%) Hispanic/Latinx, 60 (77.9%) Black, and 24 (96%) White people completed the 2-dose series. Race was not significantly associated with receiving 2 doses (*P* = .16).

Through 1 January 2023, there were 3 confirmed mpox infections among the cohort (Supplementary Table 3). All were GBSGMM and all were diagnosed at time of first mpox vaccine. Two had fever and systemic symptoms and all 3 had characteristic rashes. All were treated as outpatients (2 received tecovirimat) and made a full recovery.

## DISCUSSION

This study of 249 people receiving mpox vaccines at TOCM included 112 PLWH and 111 PLWoH. Our cohort was predominantly GBSGMM. An overall 159 (63.9%) identified as Black or Hispanic/Latinx, reflecting the largely Bronx-based population that we serve. That >10% developed a bacterial sexually transmitted infection during the study period reinforces the appropriateness of embedding mpox vaccination programs in sexual health clinics like TOCM. Our data reveal important sociostructural barriers to health care and highlight ways that our program and others like it must work to improve reach and care for the patients that we serve.

First, our data suggest that impoverished people were less likely to complete the 2-dose series, since people with private vs public insurance were 3 times more likely to receive 2 doses (adjusted odds ratio, 3.10 [95% CI, 1.28–7.49]; *P* < .01). Race/ethnicity was not significantly associated with receiving 2 doses, with 84.1% of Hispanic/Latinx, 77.9% of Black, and 96% of White individuals completing the series. Increased efforts must be made to ensure equitable access to mpox vaccination regardless of insurance type and status.

Second, while 82.7% of our cohort was established in our system, unestablished individuals had different demographics and may have been less disadvantaged. Unestablished individuals were more likely to have private insurance. Race/ethnicity was associated with whether patients were established, with significantly fewer unestablished Hispanic/Latinx and Black patients. Unestablished patients were not more likely to receive 2 doses, possibly because some received doses outside TOCM that were not available in the city registry. A minority of unestablished patients (around 5) were researchers and laboratory workers. Other unestablished individuals with better health care access may have traveled to TOCM to receive mpox vaccines due to availability.

Third, the fact that only 3 individuals in the cohort developed mpox (all diagnosed at the time of first vaccine dose) and no new cases emerged during follow-up (median, 121 days; IQR, 97–139) is potentially consistent with strong early protective immunity, though other factors may have contributed since local case counts declined rapidly [8] even as vaccination rollout was ongoing. The degree to which the new clusters of mpox infections reported in Chicago [9] and France [10] in early 2023 are due to waning immunity must be studied.

Finally, our study has limitations. Most important, missing data were not random since a higher percentage of unestablished vs established individuals had missing insurance and race/ethnicity data. Other limitations include the additional inadequacies of chart review, small sample size, and relatively short follow-up period. While our findings should be interpreted cautiously, the higher likelihood of completion of the 2-dose series for people with private vs public insurance and the different demographics of established and unestablished individuals highlight the need for increased efforts to mitigate these differences to ensure equity.

## Supplementary Material

ofad544_Supplementary_Data

## Supplementary Data

Supplementary materials are available at *Open Forum Infectious Diseases* online. Consisting of data provided by the authors to benefit the reader, the posted materials are not copyedited and are the sole responsibility of the authors, so questions or comments should be addressed to the corresponding author.

## Notes

***Author contributions*.** U. I. and E. A. M. designed the study. U. I., D. B., and E. A. M. extracted the primary data from the electronic medical record. C. M. performed statistical analysis. U. I. and E. A. M. primarily drafted the manuscript. All authors reviewed, edited and substantially participated in thecreation of the final manuscript.

***Financial support.*** None.
