# Supplementary material for: Implementation of an Mpox Vaccination Program at a Large Sexual Health Clinic in the Bronx—Lessons in Vaccine Equity
Source: Open Forum Infect Dis. 2023 Oct 31;10(11):ofad544. doi: 10.1093/ofid/ofad544 (PMC10651189; doi:10.1093/ofid/ofad544)
Supplement: ofad544_Supplementary_Data [file ofad544_supplementary_data.docx]

**Supplementary Table 1:** Characteristics of People Receiving Mpox Vaccines at a Large Sexual Health Clinic in the Bronx, NY between 8/1/2022 and 1/1/2023

| **Total (n=249)** | **People Living with HIV (n = 112)** | **People Living without HIV (n = 111)** | **People without available HIV Testing (n = 26)** |
| --- | --- | --- | --- |
| **Age^1^** |  |  |  |
| 19 - 30 | 23 (20.5%) | 41 (36.9%) | 7 (26.9%) |
| 31 - 40 | 36 (32.1%) | 38 (34.2%) | 15 (57.7%) |
| 41 - 50 | 24 (21.4%) | 19 (17.1%) | 1 (3.8%) |
| 51 - 60 | 21 (18.8%) | 13 (11.7%) | 1 (3.8%) |
| 61 - 70 | 7 (6.3%) | 0 | 2 (7.7%) |
| > / = 71 | 1 (0.9%) | 0 | 0 |
| **Self-Reported Race & Ethnicity**2 |  |  |  |
| Hispanic/Latinx | 46 (41.1%) | 34 (30.6%) | 2 (7.7%) |
| Non-Hispanic Black/African American | 46 (41.1%) | 29 (26.1%) | 2 (7.7%) |
| Non-Hispanic White | 5 (4.5%) | 14 (12.6%) | 6 (23.1%) |
| Asian | 0 | 5 (4.5%) | 2 (7.7%) |
| Native Hawaiian | 0 | 1 (0.9%) | 0 |
| Multiracial | 11 (9.8%) | 6 (5.4%) | 0 |
| Not specified/Declined | 4 (3.6%) | 22 (19.8%) | 14 (53.8%) |
| **Insurance** |  |  |  |
| Medicare | 15 (13.4%) | 4 (3.6%) | 0 |
| Medicaid | 57 (51%) | 37 (33.3%) | 5 (19.2%) |
| ADAP/PrEP-AP^3^ | 8 (7.1%) | 6 (5.4%) | 0 |
| Private Insurance | 32 (28.6%) | 56 (50.4%) | 18 (69.2%) |
| Uninsured | 0 | 3 (2.7%) | 1 (3.8%) |
| Unknown Insurance Status | 0 | 5 (4.5%) | 2 (7.7%) |
| **“Established” vs “Unestablished”** |  |  |  |
| Established | 109 (97.3%) | 95 (85.6%) | 2 (7.7%) |
| Unestablished | 3 (2.7%) | 16 (14.4%) | 24 (92.3%) |
| **Number of Doses Received** |  |  |  |
| One | 22 (19.6%) | 24 (21.6%) | 6 (23.1%) |
| Two | 90 (80.4%) | 87 (78.4%) | 20 (76.9%) |
| **Self-Reported Gender Identity:** |  |  |  |
| Cisgender man | 109 (97.3%) | 105 (94.6%) | 20 (76.9%) |
| Transgender woman | 2 (1.8%) | 3 (2.7%) | 0 |
| Genderqueer | 1 (0.9%) | 2 (1.8%) | 0 |
| Non-binary | 0 | 1 (0.9%) | 0 |
| Cisgender woman | 0 | 0 | 5 (19.2%) |
| Not specified/Declined | 0 | 0 | 1 (3.8%) |
| **Self-Reported Sexual Orientation:** |  |  |  |
| Gay | 95 (84.8%) | 94 (84.7%) | 2 (7.7%) |
| Bisexual | 9 (8%) | 9 (8.1%) | 1 (3.8% |
| Queer | 0 | 0 | 0 |
| Straight | 6 (5.4%) | 2 (1.8%) | 1 |
| Unknown/Unspecified | 2 (1.8%) | 6 (5.4%) | 22 (88.5%) |
| **Sexual Behavior:** |  |  |  |
| GBSGMM^4^ | 108 (96.4%) | 106 (95.5%) | 3 (11.5%) |
| Unspecified | 4 (3.6%) | 5 (4.5%) | 23 (88.5%) |
| **CD4 Count (cells/µL)** |  |  |  |
| <200 | 6 (5.4%) | N/A | N/A |
| >200 | 103 (92%) | N/A | N/A |
| Unknown | 3 (2.7%) | N/A | N/A |
| **HIV Viral Load (copies/mL)** |  |  |  |
| < 200 | 6 (5.4%) | N/A | N/A |
| > 200 | 101 (90.2%) | N/A | N/A |
| Unknown | 5 (4.5%) | N/A | N/A |
| **Among PLWH, ART Prescribed?^5^** |  |  |  |
|  | 111 (99.1%) | N/A | N/A |
| **Among PLWoH, PrEP Prescribed? ^6^** |  |  |  |
| TDF/FTC^7^ | N/A | 55 (49.5%) | 0 |
| TAF/FTC^8^ | N/A | 34 (30.6%) | 0 |
| CAB^9^ | N/A | 6 (5.4%) | 0 |
| None | N/A | 15 (13.5%) | 8 (30.8%) |
| Unknown | N/A | 1 (0.9%) | 18 (69.2%) |
| **Confirmed Bacterial STIs^9^** |  |  |  |
| Chlamydia | 14 (12.5%) | 22 (19.8%) | 0 |
| Gonorrhea | 21 (18.8%) | 21 (18.9%) | 0 |
| Syphilis | 15 (13.4%) | 6 (5.4%) | 0 |
| *Mycoplasma genitalium* | 0 | 3 (2.7%) | 0 |
| More than one bacterial STIs | 10 (8.9%) | 8 (7.2%) | 0 |
| None | 69 (61.6%) | 61 (55%) | 3 |
| Unknown | 4 (3.6%) | 8 (7.2%) | 23 |
| **PCR-Confirmed mpox Infection?** |  |  |  |
|  | 1 (0.9%) | 2 (1.8%) | 0 |

^1^Age = age in years at time of first mpox vaccine dose. ^2^People with more than one listed racial/ethnic identity were categorized as multiracial. ^3^ADAP = AIDS drug assistance program and PrEP-AP = PrEP patient assistance program. ^4^GBSGMM = gay, bisexual, and other sexual and gender minority men. ^5^PLWH = people living with HIV and ART = antiretroviral therapy. ^6^TDF/FTC = tenofovir disoproxil fumarate with emtricitabine. ^7^TAF/FTC = tenofovir alafenamide with emtricitabine. ^8^CAB = cabotegravir. ^9^Sexually transmitted infections (STIs) that were microbiologically confirmed in the electronic medical record between 1/1/2022 and 1/1/2023 were counted.

**Supplementary Table 2:** Logistic Regression for Characteristics Associated with Receipt of Two Vaccine Doses at a Large Sexual Health Clinic in the Bronx, NY between 8/1/2022 and 1/1/2023

|  | **OR^1^ (95% CI^2^)** | **p-value** | **aOR^3^** | **p-value** |
| --- | --- | --- | --- | --- |
|  |  |  |  |  |
| **Age** | 1.04 (1.01-1.08) | <0.01 | 1.03 (1.00-1.08) | 0.05 |
|  |  |  |  |  |
| **Age** | 1 (ref^4^) |  |  |  |
| 19-29 years | 1.85 (0.89-3.86) | 0.10 |  |  |
| 30-39 years | 2.44 (0.92-6.49) | 0.07 |  |  |
| 40-49 years | 3.40 (1.23-9.38) | 0.02 |  |  |
| >= 50 years |  |  |  |  |
|  |  |  |  |  |
| **Insurance Status^5^** |  |  |  |  |
| Public Insurance^6^ | 1 (ref) |  | 1 (ref) |  |
| Private Insurance | 3.00 (1.47-6.12) | <0.01 | 3.10 (1.28-7.49) | 0.01 |
|  |  |  |  |  |
| **Race/Ethnicity^7^** |  |  |  |  |
| Hispanic/Latinx | 1 (ref) |  | 1 (ref) |  |
| Non-Hispanic Black | 0.66 (0.30-1.48) | 0.32 | 0.69 (0.30-1.59) | 0.39 |
| Non-Hispanic White | 4.52 (0.56-36.42) | 0.16 | 2.38 (0.27-20.76) | 0.43 |
| Other | 0.60 (0.20-1.78) | 0.35 | 0.53 (0.17-1.66) | 0.28 |
|  |  |  |  |  |
| **Established vs Unestablished** |  |  |  |  |
| Established | 1 (ref) |  | 1 (ref) |  |
| Unestablished | 0.72 (0.34-1.55) | 0.41 | 0.48 (0.13-1.78) | 0.28 |

^1^OR = odds ratio. ^2^CI = confidence interval. ^3^aOR = adjusted odds ratio. ^4^ref = reference. ^5^Insurance data available for 238 individuals. ^6^Public insurance = primary insurance Medicaid, Medicare, or ADAP/PrEP-AP. ^7^Race/ethnicity data for 209 individuals.

**Supplementary Table 3:** Case characteristics of people who had a PCR-confirmed Mpox Infection and received Mpox Vaccines at a Large Sexual Health Clinic in the Bronx, NY between 8/1/2022 and 12/31/2022

|  | **Case 1** | **Case 2** | **Case 3** |
| --- | --- | --- | --- |
| **Demographics** | 47M^1^, GBSGMM^2^, PLWH^3^ | 28M, GBSGMM, PLWoH^4^ | 33M, MSM, PLWoH |
| **Signs, symptoms, and management** | - No fever or systemic symptoms  - Characteristic itchy rash with 10-20 discrete lesion, some indurated and one vesicular, distributed on upper back, and thighs  - No anal or pharyngeal pain or lesions noted  - No tecovirimat given | - Prodrome including fever, headache, lymphadenopathy, and myaglias  - Characteristic rash including some umbilicated papular lesions distributed on chest, arms, and face  - Sore throat and severe rectal pain  - Treated with tecovirimat 600 mg^5^ BID^6^ for 14 days | - Prodrome including fevers and malaise  - Characteristic rash with tender vesicular and umbilicated papular lesions distributed on face, chest, back and extremities  - Painful inguinal lymphadenopathy and clear penile discharge noted  - Treated with tecovirimat 600 mg BID for 14 days |
| **Timing of mpox diagnosis in relation to date of immunization(s)** | Received first dose at initial presentation when already symptomatic for 7 days. Received a second dose 4 weeks later after recovery | Initially declined mpox vaccine at regular visit. Developed symptoms around a week later and received a single dose of vaccine at time of mpox diagnosis (while symptomatic) | Received single dose of vaccine at time of mpox diagnosis while symptomatic |
| **Total number of mpox vaccine doses received** | 2 | 1 | 1 |

^1^M = male. ^2^GBSGMM = gay, bisexual, and other sexual and gender minority men. ^3^PLWH = people living with HIV. ^4^PLWoH = people living without HIV. ^5^mg = milligrams. ^6^BID = twice daily
